# Prevention of Diphtheria

**Published:** 1881-05

**Authors:** Edwin R. Maxson

**Affiliations:** Syracuse, N. Y.


### PREVENTION OF DIPHTHERIA.

By EDWIN R. MAXSON, A.M., M.D., LL.D., of Syracuse, N. Y.

Since diphtheria originated in Egypt, near 2,500 years ago, where it prevailed, and in Asia Minor, 500 years, before extending further, and hence was first called *Egyptian* and then *Syriac disease,* the question as to its *prevention* has often been asked, but perhaps never quite satisfactorily answered.

And yet, when the facts are carefully examined, taking into account its *history*, *causes and pathology*, there is nothing strange connected with the inquiry. A glance, then, at the subject, in a general way, on this basis, may enable us to arrive at a rational conclusion as to its *prevention*, which should be of present and future benefit to the human family.

That diphtheria, a general putrid febrile affection, so nearly allied to the plague, an offshoot of which it very likely is, should have originated, as well as the plague itself, in Egypt, from putrid animal and vegetable emanations arising from the drying up of marshes or pools of water in the old cemeteries, after the yearly overflowing of the Nile, is precisely as might have been expected. And especially when we take into account the various imprudent habits prevalent among the Egyptians during the Assyrian and Ethiopic invasion and control of the country, including numerous deviations from the laws of health and rules of propriety, many of which, with others added to the list, there and elsewhere, have been predisposing to the disease, and accounting for its spread and prevalence down to the present time.

For, though the poisonous effluvia arising from decaying animal and vegetable matters along the valley of the Nile may very likely have been the first cause of the disaster, it should be remembered that the *poison* or "bacteria," first operated upon constitutions that had been rendered imperfect, not only by their own imprudence, but also by the indiscretions of their progenitors. And the degree of physical degeneracy thus produced, together with the effect of climate, etc., in different localities, constituted the difference and predisposition of the disease. And hence it was, no doubt that diphtheria extended first into Asia Minor; where, it is likely, that all the circumstances contributed to constitute the strongest predisposition, and especially the invasion and wars of the Persians.

And the same holds true in relation to its extension, after 500 years, into the South of Europe; many degradations connected with the decline of the Roman Empire, and especially the wars with the Northern barbarians, as well as the Crusades, etc., having contributed to its extension, during the subsequent 1,500 years, across the continent of Europe, the corruptions of the dark ages having contributed to increase the predisposition to the disease and its spread.

Thus we find that in this extension, prevailing mostly in garrisoned towns, it became epidemic in Holland in A.D. 1337, in Paris in 1596, and in America in 1771, having since prevailed extensively in France, in 1818 to 1835, and in England and the United States, from 1856 to the present time. And, though the disease had originated in Egypt, no doubt, as stated, and had extended like other contagious febrile affections, in the direction most predisposing, taking all the circumstances into account, diphtheria, like all other contagious diseases, and more than many of them, as often arises spontaneously, among the predisposed, from putrid animal and vegetable substances. So that contagion alone is by no means the sole cause of the disease.

And while too much care cannot be taken to avoid exposure to the *contagion* of diphtheria, it is of as much importance as a means of exterminating it, and of vastly more importance, as a direct prevention, to remove the physical predisposition and general exciting causes of the disease as they now prevail.

The causes of diphtheria predisposing and exciting, besides contagion, may be regarded as embracing every possible deviation from the laws of health and rules of propriety, not only of the children, who are the more common victims of it, but also of their ancestors. For many children are hereditarily predisposed to this disease from the effects of diseased parents, the result of various imprudences, as the use of tobacco, intoxicating drinks to excess, licentions habits, improper food and irregular eating; and, in short, every deviation of the parents, or their progenitors, from the proper rules of life, physical, intellectual and moral.

Many children also become predisposed to diphtheria by irregular feeding in infancy, improper clothing, such as short sleeves short pants, etc., and irregularity in taking food, poisoned candies and various unwholesome and indigestible articles of food and drink during childhood; and later, improper food and unwholesome drinks, laet suppers, tobacco, etc. All these and many other kindred improprieties are allowed by too many parents, tending to impair digestion, derange the circulation, and interrupt nutrition, leading to an enfeebled state of vital energy, and hence predisposing to diphtheria.

Now add to all these influences *contagion*, and the various exposures of children, to sewer gas filthy, damp apartments, water closets, and filth in back yards, around barns, and in the streets, as well as the too frequent use of impure water, and we are left to wonder that so many escape the disease at all, or that so many recover, having contracted the disease, as do.

Diphtheria is a general putrid contagious febrile affection, more nearly allied, perhaps, to the *Plague*, than any other disease, and whether originating in contagion, or from putrid animal and vegetable decaying matters, may have an incubation of a week or longer. Like small-pox, it may be produced by the inhalation of an impalpable effluvium, or by palpable matter; in the one case developing the general febrile state, before there is any local manifestation of the disease; while in the other there is first a local disease, and then a general febrile state developed; and the *manner* of the introduction of the poison into the system appears to modify the disease, precisely as in the case of small-pox as contracted by the effluvium or innoculation.

The poison of diphtheria, whether from contagion or putrid exhalations, and however introduced into the system dissolves, more or less, the blood, rendering it unfit to sustain the nervous system; thus undermining the various functions of the body, and the partially dissolved fibrin and albumen become a more or less putrid feebly organized fibrino albuminous exudation membrane, on the surface of the fauces, larynx, trachea, bronchiæ, and sometimes on the lining membrane of the heart, arteries, veins and alimentary canal, one entire cast of which, measuring nearly a pint, having fallen under my observation in a boy that recovered.

Death may result from the mechanical effect of this membrane in the fauces, larynx, trachea, or bronchiæ, by interrupting respiration, hindering decarbonization of the blood, or it may occur from the direct depravity of the blood, failing to sustain vitality; or, again, by an exudation into or upon the brain and spinal cord or their membranes, producing mechanical pressure, and hence paralysis of the voluntary and often of the vital functions. Death may, however, occur from a combination of these various conditions, attended with general derangement before the suspension of the gastric, hepatic and renal functions, *uremic* poisoning sometimes suspending vitality.

Hence it is that all depressing influences, including every deviation from the laws of health, may not only predispose to this disease, but will, in every case, greatly increase the danger of a fatal termination when once contracted.

*Prevention.*—To prevent diphtheria, then, and so finally exterminate it, every man, woman and child throughout our land and the world, should be brought to obey the laws of life and health.

Parents should regularly feed, properly clothe and duly restrain all children before they come to the years of understanding and accountability. This alone would do much. A late prominent physician of Paris estimated that 3,000 children had died in that city, during the thirty years of his practice there, from short sleeves, short pants, and other kindred imprudence in the dressing of children. And I am fully convinced that as large a proportion are sacrificed, in towns, at least, in this country, from the same cause—all for a *wicked* fashion. And from careful observation, in this country and abroad, I am confident that at least as many more are carried off by improper food and irregularity in taking it; together with poisonous candies, and other unwholesome and indigestible trash, that no child or other person should eat.

Many of these, it is true, do not die of diphtheria. But it should be remembered that all this goes to predispose those not actually killed by depraving the blood and lessening the powers of vital resistance. And hence, when exposed to the *contagion* of diphtheria, or to putrid animal and vegetable exhalations, they are the first to take and the most liable to die of it.

Children on attaining the age of accountability, and all other persons, should take plain, nourishing and digestible food with strict regularity, and nothing between meals or late at nights. Trash, tobacco, intoxicating drinks, cosmetics, hair-dyes, dime novels, etc., should be avoided by all. And while the amount of clothing should not be in excess, care should be taken to keep the arms, legs and feet well protected, and all dress should be adapted to the season.

The person should be kept *clean*, without too much fretting the skin by unnecessary washing, lest the urinary or other excretions should be called to the surface, thereby increasing personal filth and injuriously deranging the various functions of the body. Sleeping rooms should be as far from the ground as possible; water should not be allowed in the cellar for a day even; and no decaying vegetables should be kept there.

Pure air should be allowed to pass into, and foul air out of, sleeping and all other rooms, without admitting dampness, or exposing the occupants to the chilly night air more than can be helped.

No stagnant water should be allowed about a dwelling. And the back yards, where the children play, should be kept exquisitely clean. Drains for sinks should be kept in order; and privy vaults should be cleaned out as often as twice a year lime being thrown in at least once a week, and if convenient dry earth each day.

Heaps of filth should never be allowed about barns, or other out-houses. Hencoops, pig stys and rabbit's cages, if allowed, should be as far from the house as possible, and kept exquisitely clean; and no water should be used that could possibly contain decaying animal and vegetable matter.

Children should not be put to such kind of labor as would expose them to injuries from filth, damp air, or other injurious influences. And adults should avoid such exposure as far as possible.

Now, as it was a deviation from all these rules of propriety which has predisposed to and kept up diphtheria and all other kindred diseases, it is only by a return to these laws of health and rules of propriety, in every minute particular, that they are to be prevented and exterminated. And while all this cannot be accomplished at once, very much can be done *now*, and more ultimately, by getting right in all these particulars And public hygiene, carrying out these principles in ample drainage, supplying pure water, cleaning streets, and suppressing all nuisances, may aid greatly in this work. And when a generation shall have been raised up with such habits, and without the hereditary predisposition to this and other kindred diseases, children not inheriting it, properly cared for by their parents, and obeying all the laws of life and health, may become in a great degree secure from the ravages of diphtheria, and hence of other putrid diseases. And as the body is the instrument of the mind, physical disease may not only be eradicated in the main but the intellectual and moral powers of mankind will become proportionately elevated; and thus humanity may in a measure approximate Divinity, and become more nearly "allied to angels on the better side." Let us labor for this, then, as not only involving the *physical*, but also in an equal degree the *intellectual and moral well being of mankind.—The Sanitarian.*

